# Optimization of Enzymatic Extraction Process for *Peony lactiflora* Seed and Its Application in Skin Care

**DOI:** 10.1111/jocd.70328

**Published:** 2025-10-16

**Authors:** Zhixiong Chen, Ping Cao, Yong Yang, Yaolin Wen, Shaohui Xia, Guangxi Fan, Jie Zhu

**Affiliations:** ^1^ Huzhou Jiaheng Industrial Co. Ltd. Huzhou China; ^2^ College of Landscape Architecture Beijing Forestry University Beijing China; ^3^ Catch Bio‐Science & Technology Co. Ltd. Beijing China; ^4^ Animal Husbandry and Veterinary Station of Wuzhong District Changsha China

**Keywords:** DPPH, peony seed, response surface methodology, soothing effect, water enzymatic method, whitening

## Abstract

**Background:**

The peony, as a botanical marvel, boasts a wealth of polysaccharides, total flavonoids, paeoniflorin, and other bioactive chemical constituents across all its parts. While past research has primarily concentrated on the flowers and roots of this plant, there has been a relative paucity of investigation into the seeds of Paeoniflora. This paper aimed to bridge this gap by providing a foundational study on the Paeoniflora seeds, offering valuable insights for future research endeavors in this area.

**Objective:**

(a) Optimization of peony seed extraction process; and (b) exploring the skin beautifying effects of *Paeonia lactiflora* seed extracts.

**Methods:**

One‐way optimization and response surface optimization techniques were used for enzymatic extraction of peony seed extract using DPPH free radical scavenging rate as an evaluation index. Tyrosinase activity inhibition and hyaluronidase inhibition were determined in vitro under optimal extraction conditions.

**Results:**

The results showed that the maximum DPPH radical scavenging rate of 86.94% ± 1.02% was obtained experimentally under the simulated optimal conditions of 60°C, 0.010 (g/g) solid–liquid ratio, 3.9% (g/g) addition of complex enzyme, 0.39 (g/g) cellulose ratio, and 2.7 h enzymatic hydrolysis time. Meanwhile, the inhibition rate of tyrosinase activity and hyaluronidase inhibition rate were determined under the optimal extraction conditions. The results showed that the inhibition rate of tyrosinase was 61.4% and that of hyaluronidase was 77.7% in the concentration range of 0.5%–2.5%.

**Conclusions:**

The complex enzymatic method is an effective method for the production of peony seed extract with significant antioxidant properties, whitening activity, and soothing effect.

## 1 Introduction

Peony ( *Paeonia lactiflora* Pall.) is a perennial herbaceous plant belonging to the peony family, with over 4000 years of cultivation history in China, possessing high ornamental [1] and medicinal value [2]. To date, hundreds of varieties have been cultivated, primarily focusing on the ornamental characteristics of the flowers, which do not hold significant value for oil production. The seeds of peony species have considerable oil value, and with peony seed oil being classified as a new resource food, research into the oil value of peony plants has deepened [3]. Currently, most research on peony has concentrated on its roots and flowers, with limited development and utilization of the seed resources, despite the presence of many substances beneficial to human health in the seeds [4]. Peony seeds contain unsaturated fatty acids (such as oleic acid, linoleic acid, and alpha‐linolenic acid), trace mineral elements (such as Ca^2+^ and Mg^2+^), and other active substances (such as phytosterols, tocopherols, and squalene), which have certain health and nutritional benefits [5] and have been widely applied in the fields of food, medicine, and cosmetics [6].

Peony, as one of the flowers, contains rich polysaccharides, total flavonoids, paeoniflorin, and other chemical components in all parts. Its peony flower extract has been listed in the catalog of cosmetic raw solids used (2021 edition). Zhou [7] identified 27 chemical components in peony using ultra‐high‐performance liquid chromatography‐quadrupole time‐of‐flight mass spectrometry. Both the water extract and the ethanol extract exhibited antioxidant activity against DPPH and ABTS, as well as inhibitory effects on enzymes such as TYR、PLPF and its main compounds had broad development prospects as new drug and cosmetic formulations. Yan [8] studied the fatty acids, total phenol content, and total flavonoid content of 17 peony varieties' seeds, and found that peony seed extract has good antioxidant properties in total antioxidant capacity and free radical scavenging rate. Chen [9] studied the *P. lactiflora* pall seeds oil, and found that it has whitening, anti‐inflammatory, and anti‐aging properties.

The plant cell wall, a complex composite primarily consisting of cellulose, hemicellulose, and pectin, forms an intricate three‐dimensional network that serves as a natural barrier to the release of encapsulated active ingredients [10]. Cellulase enzyme predominantly catalyzes the hydrolysis of β‐1,4‐glucoside bonds [11], thereby disrupting the structural integrity of the cellulose main chain. Concurrently, pectinase enzyme is responsible for the degradation of ester bonds and alpha‐1,4‐glucoside bonds present in pectin polysaccharides, which contributes to the softening of intercellular junctions. The complementary action of cellulase and pectinase elicits a pronounced “synergistic effect,” wherein cellulase initiates the fragmentation of the cell wall, creating discrete particles [12]. Subsequently, pectinase acts to further disassemble the adhesive interactions between these fragments, thereby enhancing the liberation of cellular contents. Utilizing a complex enzyme mixture for the enzymatic hydrolysis of *Paeonia* seeds has resulted in the production of a higher concentration of active ingredients. This extraction methodology represents an innovative approach in the field. The present study has demonstrated that the extract obtained from the complex enzyme enzymatic hydrolysis of *Paeonia* seeds exhibits notable skincare properties. These findings provide a valuable technical reference for the development and effective application of natural plant extracts within the cosmetics industry, potentially advancing the utilization of such bioactive compounds for dermal health.

The present study evaluated the DPPH free radical scavenging activity of peony seed extract. Using one‐way and response surface methodology, the study provided insights into the effects of various factors such as temperature, solid–liquid ratio, enzyme dosage, cellulase ratio, and duration on the ability of peony seed extract to neutralize DPPH free radicals [13]. In conjunction with this, the research also examined additional in vitro skincare benefits of the extracts produced under the optimal enzymatic hydrolysis conditions.

## 2 Materials and Methods

### 2.1 Main Materials and Reagents

*P. lactiflora* seed (Tibet); α‐Arbutin (98%), Shanghai Yien Chemical Technology Co. Ltd.; Pectinase (100 U/mg), Cellulase (50 U/mg), P‐DAB, hyaluronidase and tyrosinase, Beijing Solarbio Technology Co. Ltd.; Sodium hydroxide and hydrochloric acid (Guangdong Guanghua Technology Co. Ltd.); DPPH detection kit, Nanjing Jiancheng Bioengineering Institute; other reagents, analytical grade.

### 2.2 Main Instruments and Equipments

Swing high‐speed grinder DFY‐500C, Lintech Machinery Co. Ltd. in Wenling City; Precision balance Mettler ME204E, Shanghai Zhuohao Technology Co. Ltd.; Electric constant temperature water bath pot DHP‐9082B, Shanghai Yiheng Technology Co. Ltd.; Rocking bed SK‐0180‐S, Dalongxingchuang Experimental Instrument (Beijing) Co. Ltd.; Multi‐function microplate reader MULTISKAN, purchased from Thermo Fisher Scientific Co. Ltd.; TDL‐60C low‐speed centrifuge, Shanghai Anting Scientific Instrument Factory.

### 2.3 Experimental Methods

#### 2.3.1 Enzymatic Extraction Process of Peony Extract

The peony seed extract extraction process includes: peony seeds → crushing → mixing and homogenization with water → pH adjustment → complex enzyme treatment → enzyme inactivation → centrifugation → collection of supernatant → determination of DPPH removal rate.

#### 2.3.2 Single‐Factor Experiment

One‐way experiments were carried out to investigate the effects of extraction conditions such as enzymatic temperature (A), solid–liquid ratio (B), addition of composite enzyme (C), cellulase ratio (D), and enzymatic time (E) on the free radical scavenging ability of *P. lactiflora* seed extract on DPPH. The effects of different temperatures 45°C, 50°C, 55°C, 55°C, and 60°C on the removal of DPPH by *P. lactiflora* seed extract were investigated by fixing the solid–liquid ratio of 0.02 (g/mL), the addition of 3% (g/mL) of composite enzyme, the proportion of cellulase 0.4 (g/g) and the enzymatic time of 2.5 h. The experiments were conducted in triplicate to investigate the impact of varying solid–liquid ratios, specifically 0.01, 0.02, 0.03, 0.04, and 0.05 (g/mL), on the DPPH radical scavenging activity of peony seed extract. Each ration was tested three times to ensure the reliability and concictency of the results. The fixed temperature of 45°C, the solid–liquid ratio of 0.02 (g/mL), the cellulase ratio of 0.4 (g/g), the enzyme digestion time of 2.5 h, to investigate the effect of different composite enzyme additions selected as 1%, 2%, 3%, 4%, 5% (g/mL) on the removal rate of DPPH by *P. lactiflora* seed extract. Repeated three times; fixed temperature of 45°C, solid–liquid ratio of 0.02 (g/mL), 3% (g/mL) composite enzyme addition, enzyme digestion time of 2.5 h, to investigate the effect of different cellulase ratios added proportion of 0, 0.2, 0.4, 0.6, 0.8, 1.0 (g/g) on the removal rate of *P. lactiflora* seed extract on DPPH, repeated three times; fixed temperature 45°C, solid–liquid ratio 0.02 (g/mL), 3% (g/mL) composite enzyme addition, cellulase proportion 0.4 (g/mL), to investigate the effect of different enzyme digestion time selections of 1.5, 2, 2.5, 3, 3.5 h on the removal rate of DPPH by *P. lactiflora* seed extract, repeated three times. The above conditions were chosen as a single factor for experimental validation.

#### 2.3.3 DPPH Free Radical Scavenging Ability Measurement

This was performed according to the method referenced in literature [14]. One milliliter sample of peony seed extract solution was taken in a test tube, and 2 mL of 0.04 mg/mL DPPH ethanol solution was added. The mixture was shaken and allowed to stand for 30 min, after which the absorbance (*A*_*i*_) was measured at a wavelength of 517 nm. Anhydrous ethanol (1 mL) was used as a blank control, and its absorbance (*A*_0_) was also measured at 517 nm. The DPPH free radical scavenging rate was calculated using Equation (1) [15]:

(1) $$\text{The clearance of DPPH}\%=\left[1-\left(A_{0}-A_{i}\right)/A_{0}\right]\times 100\%$$

#### 2.3.4 Response Surface Optimization Experiment

Based on the single‐factor experiments, a Box–Behnken central composite design was used to design the response surface experiment. The levels of experimental factors are shown in Table 1.

**TABLE 1:** Experimental design of response surfaces.

Level	Factor				
	Temperature (°C)	Solid–liquid ratio (g/mL)	Enzyme amount (%) (g/mL)	Cellulase ratio (%) (g/g)	Time (h)
−1	50	0.01	2	0.2	2
0	55	0.02	3	0.4	2.5
1	60	0.03	4	0.6	3

#### 2.3.5 Tyrosinase Inhibition Experiment

The optimal enzymolysis conditions obtained by response surface experiment were used to obtain the extract solution of peony and dilute it into five concentrations of 0.5%, 1%, 1.5%, 2%, and 2.5% for the determination of tyrosinase inhibition rate. 1.50 mmol/L l‐tyrosine solution and 50.00 U/mL tyrosinase solution were prepared. In the test tube, 1.00 mL *Paeonia* extract solution, 1.00 mL PBS, and 0.5 mL tyrosinase were added as the experimental group. 1.00 mL sample solution and 1.5 mL PBS were added as the blank group. 2.00 mL PBS and 0.5 mL tyrosinase were added as the control group. 2.5 mL PBS buffer was added as the control blank group. Then, the four groups of tested solution were placed at 37°C for 10 min, and 1.00 mL l‐tyrosine solution was added, respectively, and the absorbance at 475 nm was measured after the reaction at 37°C for 10 min. Phenylethylresorcinol and α‐Arbutin were used as the control group. l‐tyrosine (monophenolase) inhibition rate (%) was calculated according to Equation (2) [16]:

(2) $$\text{Inhibition rate}=\left[1-\left(D_{1}-D_{2}\right)/\left(C_{1}-C_{2}\right)\right]\times 100\%$$

where *D*_1_, *D*_2_, *C*_1_, and *C*_2_ are the absorbance of the experimental group, experimental blank group, control group, and control blank group, respectively.

#### 2.3.6 Hyaluronidase Inhibition Rate Test

To prepare P‐DAB solution, 4 g P‐DAB was weighed, and 50 mL 10 mol/L HCl, mix and 350 mL iced vinegar sour were added. 50 μL *Paeonia* extract solution was taken, and 50 μL 7900 U/mL hyaluronidase, 50 μL 12.5 mmol/L calcium chloride, and 250 μL 1.2 mg/mL sodium hyaluronate were added and boiled in a 37°C water bath for 20 min, and for 40 min. 50 μL 0.4 mol/L NaO, and 100 μL 0.2 mol/L sodium borate were also added to the water bath and for 3 min. The solution was removed from the water bath and cooled to room temperature. 1.5 mL P‐DAB solution was added, and the absorbance was measured at 585 nm after 20 min in a 37°C water bath. For the blank group, peony extract solution was replaced with water and for the positive group, 1.0 mg/mL dipotassium glycyrrhizinate was replaced with water. The calculation formula of hyaluronidase inhibition rate is shown in Equation (3) [9]:

(3) $$\text{Hyaluronidase inhibition rate}=\left(A_{1}-A_{2}\right)/A_{1}\times 100\%$$

where *A*_1_ and *A*_2_ are the absorbance of the blank group and the experimental group, respectively.

#### 2.3.7 Data Processing

Analysis of variance (ANOVA) and multiple regression analysis were conducted for fitting the model using Box–Behnken design. The data were evaluated with various statistical analysis parameters such as *p* value, *F* value, degrees of freedom (*D*_*f*_), sum of squares (SS), mean sum of squares (MSS), coefficient variation (C.V.), absolute average relative deviation (AARD), determination coefficient (*R* ^2^), and adjusted determination of coefficient ($R_{\mathrm{adj}}^{2}$), correlation coefficient (*R*) to reproduce the statistical significance of the developed quadratic mathematical model. Data processing and significant difference analysis and plotting were carried out using Graphpad prism 8.0; response surface experimental design and analysis were performed using the Design Expert software.

## 3 Results

### 3.1 Effect of Single Factor in DPPH Removal From Peony Seed Extracts

#### 3.1.1 Effect of Temperature on the Removal of DPPH by Peony Seed Extracts

**FIGURE 1:**
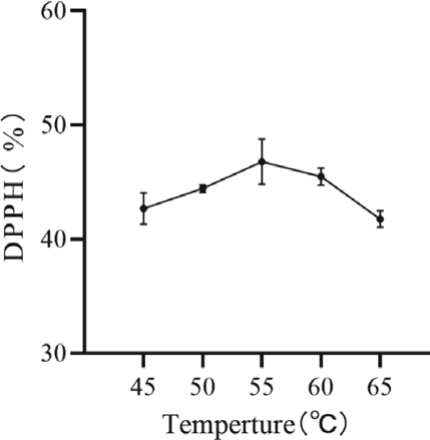
Effect of temperature on the removal of DPPH by peony seed extracts.

As shown in Figure 1, DPPH removal first increased and then decreased with increasing temperature. The highest DPPH removal was observed at a temperature of 55°C. The experiments showed that there is an optimum temperature interval when cellulase is coupled with pectinase, allowing the enzyme activity to be fully released [17]. Therefore, temperatures of 50°C, 55°C, and 60°C were selected for the response surface optimisation tests.

#### 3.1.2 Effect of Solid–Liquid Ratio on the Removal of DPPH by Peony Seed Extracts

**FIGURE 2:**
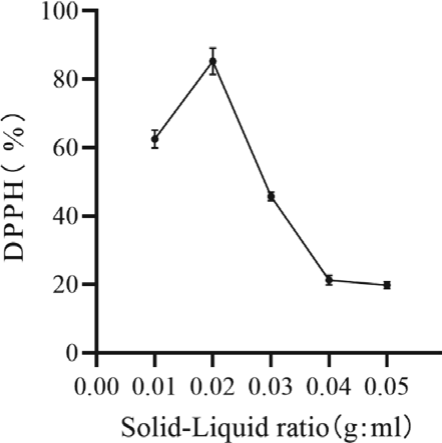
Effect of solid–liquid ratio on the removal of DPPH by peony seed extracts.

As shown in Figure 2, DPPH removal first increased and then decreased with increasing material‐liquid ratio. The highest DPPH removal was observed at a material‐liquid ratio of 1:100 (g/mL). An excessively low solid‐liquid ratio can lead to inadequate dissolution of the substance, whereas an overly high solvent volume may cause the excessive dissolution of impurities, potentially compromising the purity of the solution. This affects the effect of peony seed extract on DPPH removal [18]. Therefore, material–liquid ratios of 0.01, 0.02, and 0.03 (g/mL) were selected for response surface optimization experiments.

#### 3.1.3 Effect of Composite Enzyme on the Removal of DPPH by Peony Seed Extracts

**FIGURE 3:**
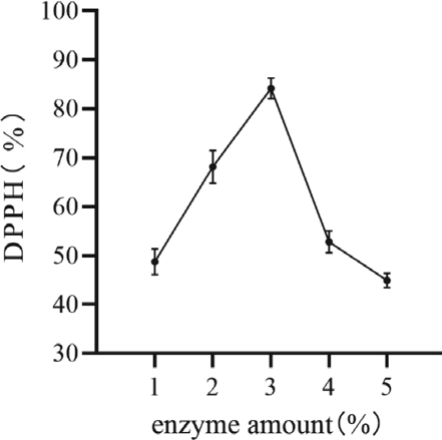
Effect of composite enzyme on the removal of DPPH by peony seed extracts.

The DPPH removal rate initially increased and then decreased with the increase of enzyme addition (Figure 3). The highest DPPH removal was observed when the amount of composite enzyme added was 3% (g/mL). At higher composite enzyme addition, the dissolution of the active ingredient was promoted; with the increase of composite enzyme addition beyond the optimum addition, a large amount of substances in the peony seeds were dissolved, which affected the active ingredient, resulting in a decrease in the DPPH removal rate [19]. Therefore, the material–liquid ratios of 2%, 3%, and 4% (g/mL) were selected for the response surface optimization test.

#### 3.1.4 Effect of Cellulase Ratio on the Removal of DPPH by Peony Seed Extracts

**FIGURE 4:**
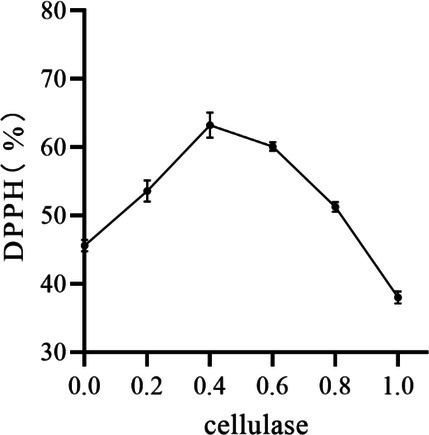
Effect of cellulase ratio on the removal of DPPH by peony seed extracts.

As shown in Figure 4, DPPH removal first increased and then decreased with increasing cellulase percentage. The highest DPPH removal was observed at a cellulase percentage of 0.4 (g/g). This may be due to the fact that the enzyme molecules involved in both the pectin reaction and the cellulose hydrolysis reaction in the hydrolysis environment reached the steady state of the enzyme‐binding reaction system at a cellulase percentage of 0.4 (g/g) [20]. Therefore, cellulase percentages of 0.2, 0.4, and 0.6 (g/g) were selected for the response surface optimisation test.

#### 3.1.5 Effect of Enzymatic Time on the Removal of DPPH by Peony Seed Extracts

**FIGURE 5:**
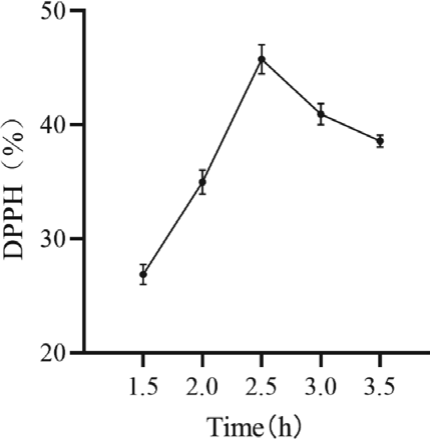
Effect of enzymatic time on the removal of DPPH by peony seed extracts.

As illustrated in Figure 5, the DPPH removal rate exhibited a biphasic response to enzyme digestion time. Initially, an increase in digestion time led to a rise in removal efficiency. However, beyond a certain threshold, the removal rate began to decline. At enzyme digestion times of < 2.5 h, the DPPH removal rate demonstrated an increasing trend, with a more pronounced degree of change. All antioxidant substances were extracted in their entirety at 2.5 h. With the extension of time, the dissolution of other substances may potentially inhibit the DPPH removal rate [21]. Consequently, the enzyme digestion times of 2.0, 2.5, and 3 h were selected for the response surface optimization test.

### 3.2 Response Surface Model Fitting and Data Analysis

The experimental data from Table 1 were subjected to regression fitting and variance analysis, and the results are shown in Table 2. Based on the final equation of coded factors, we obtained DPPH (%) = 61.09 + 6.08 *A* + 19.03 *B* − 1.80 *C* + 4.32 *D* + 0.1581 *E* − 18.19 *AB* + 16.27 *AC* + 1.53 *AD* − 5.11 *AE* − 20.21 *BC* − 0.5325 *BD* − 14.84 *BE* − 1.58 *CD* − 4.35 *CE* − 1.96 *DE* − 1.33 *A* ^2^ − 5.69 *B* ^2^ − 6.11 *C* ^2^ − 5.64 *D* ^2^ − 3.04 *E* ^2^.

**TABLE 2:** Shows the variance analysis of the regression equation for the response surface optimization experiment.

Source	Sum of squares	*D*_*f*_	Mean square	*F*‐value	*p*	Distinctiveness
Model	12 453.97	20	622.70	4.53	0.0003	Significant
*A*‐temperature	592.07	1	592.07	4.31	0.0484	*
*B*‐solid–liquid ratio	5794.25	1	5794.25	42.14	< 0.0001	**
*C*‐enzyme amount	52.02	1	52.02	0.3783	0.5441	
*D*‐cellulase ratio	297.99	1	297.99	2.17	0.1535	
*E*‐time	0.4001	1	0.4001	0.0029	0.9574	
*AB*	1324.23	1	1324.23	9.63	0.0047	**
*AC*	1058.20	1	1058.20	7.70	0.0103	*
*AD*	9.42	1	9.42	0.0685	0.7956	
*AE*	104.35	1	104.35	0.7589	0.3920	
*BC*	1633.37	1	1633.37	11.88	0.0020	**
*BD*	1.13	1	1.13	0.0082	0.9284	
*BE*	880.31	1	880.31	6.40	0.0181	*
*CD*	9.95	1	9.95	0.0724	0.7901	
*CE*	75.60	1	75.60	0.5498	0.4653	
*DE*	15.33	1	15.33	0.1115	0.7413	
*A* ^2^	15.43	1	15.43	0.1122	0.7404	
*B* ^2^	282.60	1	282.60	2.06	0.1641	
*C* ^2^	325.41	1	325.41	2.37	0.1365	
*D* ^2^	277.24	1	277.24	2.02	0.1680	
*E* ^2^	80.90	1	80.90	0.5883	0.4502	
Residual	3437.51	25	137.50			
Lack of fit	2552.52	20	127.63	0.7211	0.7287	Not significant
Pure error	884.99	5	177.00			
Cor total	15891.48	45				

** *p* < 0.01.

* *p* < 0.05.

The regression model (*p* < 0.0001) is significant, and the lack of fit (*p* > 0.05) is not significant, indicating that this model is reliable for analyzing and predicting results. Additionally, the regression coefficient *R* ^2^ = 0.7837 and adjusted determination co‐efficient $R_{\mathrm{adj}}^{2}$ = 0.6106 [22], with a precision of 10.04 and a coefficient of variation (C.V) of 21.91%, indicate that the obtained regression equation has good reliability and stability, allowing for effective analysis of the DPPH scavenging rate (%) of peony seed extracts against various response values.

From Table 2, it can be concluded that the *p*‐values for temperature and solid‐to‐liquid ratio are both < 0.05, indicating that these two factors significantly affect DPPH. Based on the magnitude of the *p*‐values, the two factors with the greatest impact on the DPPH free radical scavenging ability of peony are the solid‐to‐liquid ratio and temperature, with the solid–liquid ratio (*B*) having a greater influence than temperature (*A*). The *p* values for the interaction terms *AB*, *AC*, *BC*, and *BE* are all < 0.05, indicating significant differences, with the interaction strength ranked as *BC* > *AB* > *AC* > *BE*. The values for *AD*, *AE*, *BD*, *CD*, *CE*, and *DE* are < 0.05, indicating no significance; the quadratic terms *A* ^2^, *B* ^2^, *C* ^2^, *D* ^2^, and *E* ^2^ also show no significant differences (*p* > 0.05) [23].

### 3.3 Interaction Analysis of Response Surface Optimization for DPPH Scavenging by Peony

**FIGURE 6:**
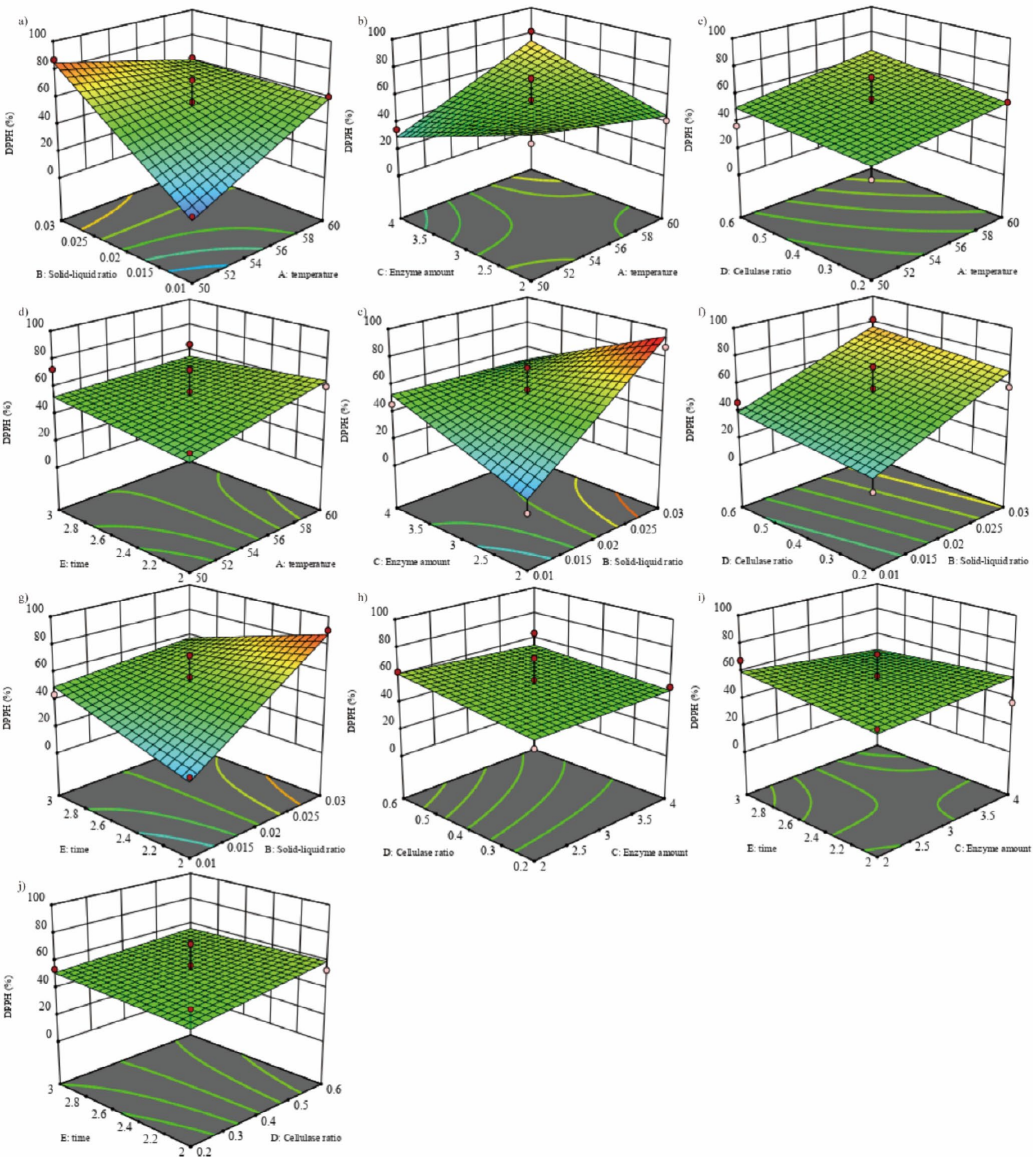
Effects of the interaction of temperature, solid–liquid ratio, enzyme amount, cellulose ratio, and time on the DPPH free radical removal rate.

Figure 6 presents the 3D surface plots of the interactions between temperature, solid‐to‐liquid ratio, enzyme amount, cellulose ratio, and enzymatic hydrolysis time. The study indicates that when the surface trend of the 3D plot is steep [24], the interaction has a significant impact on DPPH. The highest point on the 3D plot represents the optimal state of the interacting factors. From Figure 6, it can be observed that the response surface plots for *AB*, *AC*, *BC*, and *BE* show steep trends, with the steepness of the *BC* curve being greater than that of the other groups, indicating that the *BC* interaction has the greatest impact on DPPH. The interactions of *AD*, *AE*, *BD*, *CD*, *CE*, and *DE* have relatively smaller effects on DPPH. The specific interaction strength is ranked as *BC* > *AB* > *AC* > *BE* > *AE* > *CE* > *DE* > *CD* > *AD* > *BD*, consistent with the results of the variance analysis of the regression equation in Table 2.

### 3.4 Verification of Optimal Conditions

**FIGURE 7:**
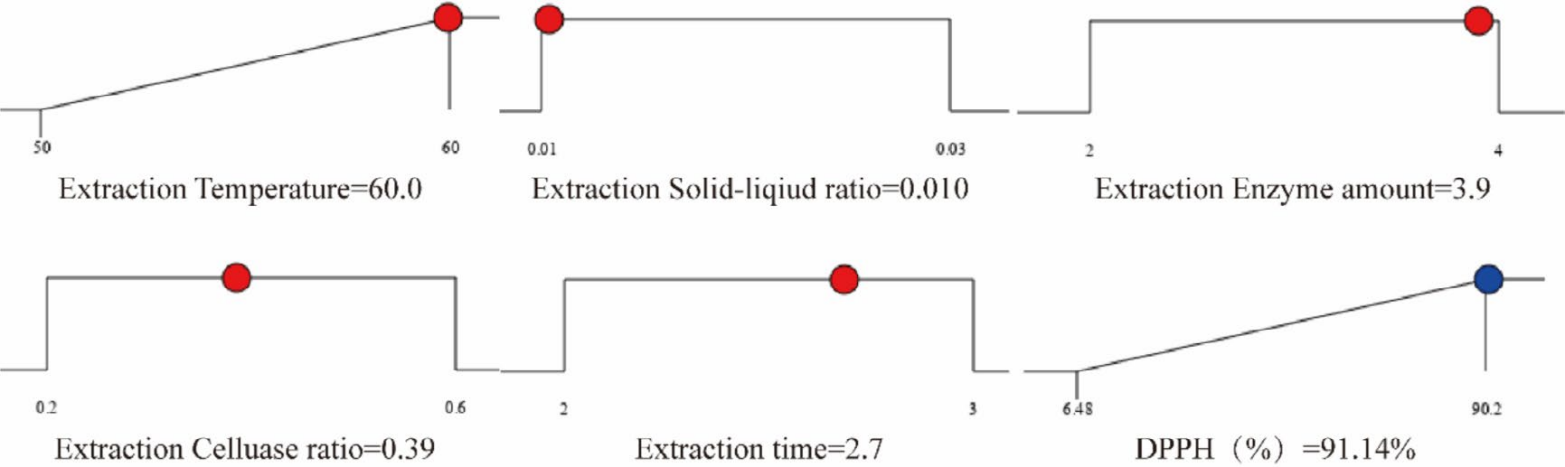
Desirability ramp for optimization.

The optimal process conditions for extracting peony seed extracts, determined through response surface experiments, are a temperature of 60°C, a solid‐to‐liquid ratio of 0.010 (g/mL), a composite enzyme addition of 3.9% (g/mL), a cellulose ratio of 0.39 (g/g), and an enzymatic hydrolysis time of 2.7 h. Under these conditions, the predicted DPPH free radical scavenging rate is 91.14%. A desirability slope was established from optimal points through a numerical optimization technique, as shown in Figure 7.

The experiments were validated in triplicate using the optimum additions mentioned above [25]. The optimized values of these tested parameters were validated under similar conditions (*n* = 6) and the average removal of DPPH under optimized extraction conditions was 86.94% ± 1.02%, with a validity of 95.10%. The results of the analysis confirmed that the response model was sufficient to reflect the expected optimization and that the model was satisfactory and accurate. The analytical results also closely matched the data obtained from the optimization using the willingness function, indicating that the Box–Behnken design can be effectively used to optimize the DPPH removal parameters of peony seed extract.

### 3.5 Whitening Effect of Peony Seed Extract In Vitro

**FIGURE 8:**
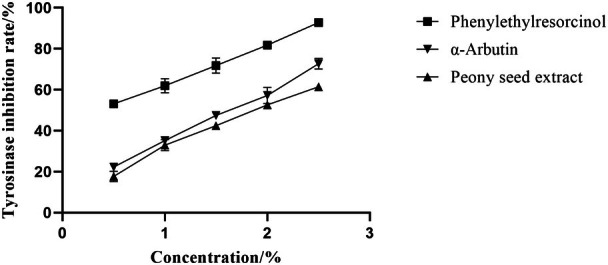
Inhibition rate of peony seed extract on tyrosinase monophenolase.

Tyrosinase is a pivotal enzyme involved in the synthesis of melanin within the cell, and a whitening effect can be realized by inhibiting its activity to impede pigmentation [25]. Figure 8 illustrates a dose‐dependent increase in the inhibition rate of tyrosinase monophenolase as the concentration of peony seed extract was elevated. Notably, at a concentration of 2.5%, the peony seed extract achieved an inhibition rate of 61.4%. In comparison, the control group, treated with phenylethylresorcinol, exhibited the highest inhibition rate at 92.8%, while α‐arbutin demonstrated a tyrosinase inhibition rate of 72.7%. The findings of this study indicate that, while peony seed extract exerts a certain degree of inhibitory action on tyrosinase, its effectiveness is comparatively inferior to that of conventional whitening agents. The reduced activity of the peony seed extract may be attributed to its complex composition and lower purity. Consequently, subsequent experiments could focus on optimizing the extract's composition through separation and purification processes to enhance its whitening potential.

### 3.6 The Soothing Skin Care Effect of Peony Seed Extract In Vitro

**FIGURE 9:**
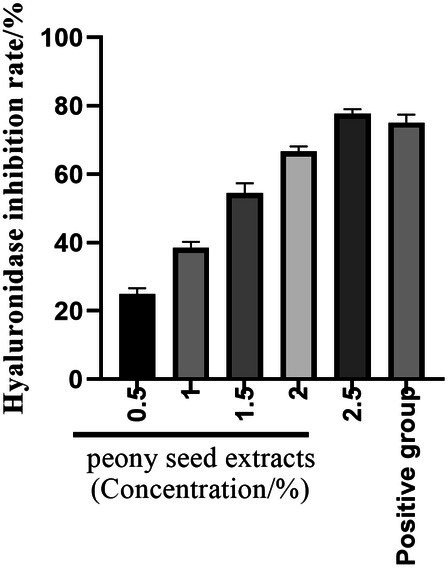
Hyaluronidase inhibition rate of peony seed extract.

Hyaluronidase plays a significant role in inflammation and allergic responses, being intricately involved in type I allergic reactions [26]. This enzyme has the ability to break down hyaluronic acid (HA) within the body, transforming it into a low molecular weight acidic irritant that can trigger the release of histamine and elicit sensitive symptoms. Consequently, the soothing properties of cosmetic ingredients can be assessed through in vitro tests that measure their inhibitory effect on hyaluronidase. Figure 9 reveals that the peony seed extract exhibits a dose‐dependent increase in its inhibitory rate on hyaluronidase within the concentration range of 0.5%–2.5%. At the highest tested concentration of 2.5%, the extract achieved an inhibition rate of 77.7% against hyaluronidase, which is comparable to the effect of 1.0 mg/mL dipotassium glycyrrhizinate. This finding suggests that peony seed extract is capable of significantly suppressing hyaluronidase activity, thereby demonstrating its potential soothing effect on the skin.

## 4 Discussion

In the one‐way result experiment, it was found that the temperature of 50°C, 55°C, and 60°C, the solid–liquid ratio of 0.01, 0.02, 0.03 (g/mL), the amount of composite enzyme of 2%, 3%, 4% (g/mL), the proportion of cellulase of 0.2, 0.4, 0.6 (g/g), and the enzyme digestion time of 2, 2.5, and 3 h were selected [27, 28, 29]. Combined with the response surface analysis, the interactions between temperature and amount of composite enzyme, and solid–liquid ratio with temperature, amount of composite enzyme and time were relatively stronger, and the corresponding response surfaces were relatively steeper. The corresponding response surfaces were steeper [30].

In this study, based on the results of one‐way experiments, the Box–Behnken model was used to optimize the extraction process of *Paeonia lactiflora* seed extract, and it was found that a temperature of 60°C, a solid–liquid ratio of 0.010 (g/mL), a composite enzyme additive amount of 3.9% (g/mL), a cellulose ratio of 0.39 (g/g) and an enzyme hydrolysis time of 2.7 h were the ideal conditions for the extraction process. Under these conditions, the maximum DPPH radical scavenging rate of the extract was 86.94% ± 1.02%. The good agreement with expectation indicated that the established model was reliable and that this extraction process was stable and reasonable, which was a feasible method for extracting peony seed extract.

Further experiments were conducted to evaluate the inhibitory effects of peony seed extract on tyrosinase and hyaluronidase activities. The results showed that the extract inhibited tyrosinase activity by 61.4% and hyaluronidase activity by 77.7% in the concentration range of 0.5%–2.5%. These results support the hypothesis that peony seed extract has whitening and soothing skin care properties.

The current investigation has certain limitations, as it is primarily focused on in vitro enzymatic analyses. Future research endeavors will aim to address these shortcomings by integrating data from both in vivo and in vitro human skin models to provide a more comprehensive understanding. The paeoniflorin in peony seed extract will also be purified in subsequent experiments to investigate the effect of peony seed paeoniflorin content on skin care.

## 5 Conclusion

It can be concluded that the complex enzyme extraction method is an efficacious approach for obtaining peony seed extracts with notable antioxidant properties, whitening activity, and a soothing effect. This study provides a theoretical foundation for the utilization of peony seed extracts in the development of cosmetics.

## Author Contributions

Zhixiong Chen designed the experiments and improved the paper. Ping Cao, Yong Yang, Yaolin Wen performed the experiments. Shaohui Xia, Guangxi Fan, Jie Zhu analyzed the data.

## Conflicts of Interest

The authors declare no conflicts of interest.

## Data Availability

We declared that materials described in the manuscript, including all relevant raw data, will be freely available to any scientist wishing to use them for non‐commercial purposes.
